# Music and Medicine: being in the moment

**DOI:** 10.15694/mep.2018.0000197.1

**Published:** 2018-09-05

**Authors:** Tim Dornan, Martina Kelly

**Affiliations:** 1 Queen's University Belfast; 2 University of Calgary

**Keywords:** Music, medicine, medical humanities

## Abstract

This article was migrated. The article was marked as recommended.

This article reviews the relationship between music and medicine, informed by our own personal experiences, and by leading scholars who have opened up music and medicine for critical reflection. Performing music, we suggest, is a state of being, in the moment, with fellow musicians and audience members. This establishes bidirectional communication, which can transport both parties to better places. Medicine is, likewise, an act of being with patients, whether or not performing a technical act (a clinical procedure, for example) is part of the interaction. Subjective, non-verbal, dimensions of the interaction engage both parties’ senses. Good doctors, like good musicians, tune in to patients at a personal level. The limited research that has examined the relationship between music and medicine shows that music can help students develop auditory skills. Of potentially greater interest is the existential contribution music could make to medical education. We suggest that this could help students and doctors reflect on their experiences of being in the world, and how shared experience can relieve suffering.

## Introduction

William Osler, who is remembered as a physician of the highest stature and a great humanist, said doctors should be well versed in the classics; he also said that good medical practice is both an art and a science. ( Bliss, 1999) The link between the classics and the humane practice of medicine was so self-evident to Osler that he felt no need to justify his advocacy for medical humanities in fine detail. In the century since Osler’s death, however, science has made unimaginable progress. Cost-effective attainment of measurable outcomes is the order of the day. The case for including humanities in medical curricula is still made but now coupled with more detailed justifications. ( Charon, 2017; Kumagai, 2017) Humanities, the arguments go, will provide foundations for effective doctoring. ( Charon, 2017) They will teach tolerance of complexity and ambiguity. ( Charon, 2017; Kumagai, 2017) They will help doctors acknowledge aspects of patients’ lives that, whilst apparently tangential to medical care, show what patients value about themselves. ( Charon, 2017) They will help doctors focus on what is singular and unique rather than what is general. ( Charon, 2017; Kumagai, 2017) Humanities, according to these arguments, are a means to curricular ends.

It is unsurprising that today’s advocacy for humanities should be expressed in instrumental terms. The ‘fate of our times’ ( Frank, 2013) is for persuasive arguments to take the form: ‘we should do A because it will reliably achieve desirable and measurable outcome B’. But might this do humanities an injustice? Is there a danger that making them part of a curriculum toolkit will set them up to fail? What curriculum designs and pedagogies best yield the benefits of medical humanities? How will curriculum leaders address the scepticism of teachers whose social capital is invested in biomedical science and evidence-based practice? And, perhaps most important of all, what are the potential unintended consequences of making humanities tools to curriculum ends? Advocacy for humanities, these arguments suggest, might benefit from taking a step back and asking some fundamental questions.

Conversations about medical humanities refer to music less often than to other artforms. Perhaps that is because our culture ‘is dominated by the visual and the verbal’. ( Storr, 1992) Music is less conspicuously relevant. And yet many doctors are musicians and music therapy is a well-established practice so it would be strange to leave music out of the conversation. This article examines, at a rather fundamental level, what music is, what medicine is, what the two have in common, and how insights from music could enrich medical practice. We have stepped back from the dominant contemporary lines of argument by considering music and medicine from an existential rather than an instrumental position. Osler, we suggest, did the same. We consider what music can offer the practice of ‘being with’ sick people, as opposed to the practice of ‘doing things to’ sick people. As befits our topic, we draw on ‘narrative knowledge’ of the arts more than ‘paradigmatic knowledge’ of the sciences. ( Dornan and Kelly, 2017) We use fragments of our experiences, as physicians, of music and medicine to support our reasoning, and quote liberally from authors who have shaped the way we think about it. Sadly, the written medium forces us to contradict our own argument - it forces us to use the propositional logic of written language to express what cannot be expressed verbally. Perhaps you could compensate for that by reflecting on what we have written as you listen to a favourite piece of music.

## Music: Martina’s story

*Why am I drawn to this topic, when I can’t read a note of music? I can best answer this by sharing my experience of music as a listener. If I close my eyes I can sense the atmosphere before the concert begins, enveloped in the buzz of anticipation. The hum of genteel murmurs and instruments warming up. I like the scratchy sounds and disharmony. A momentary silence as the conductor raises his baton, a collective inhalation. The exhalation of opening notes. It takes time to settle. I’m thinking of other things, my mind contemplating work, patients or family stuff. My eyes are preoccupied too - watching other people, the musicians, trying to interpret their body language etc. But at some stage I stop thinking and just feel. I just go with it.. and that is my favourite part..when I just sense.. I see, I hear, I listen, I pulse with the music. I feel it in my body, in my pores, in my brain and something lets go. All my senses are engaged, my body is alert. I don’t understand it intellectually, but I understand it.*

## Music: Tim’s story

*As one of 100 orchestral players, I am playing Gustav Mahler’s second symphony. Some players are good friends. Some are nodding acquaintances. Some I know only by sight. Over three days, a conductor has used his artistry, passion, and humorous chiding to form us into a living, breathing, feeling whole. All our senses - indeed, our whole beings - are attuned so we can harmonise and play intricate rhythms as one.*

*‘Mahler 2’ runs for 80 minutes without a break, using a rich palette of sometimes achingly beautiful musical sound. Now, we are playing to an audience of 800 silent, but palpably present listeners. We are close to the end of the performance. The orchestra falls silent and, after a moment of suspense, a huge chorus of singers make their entry in hushed tones. Voices and orchestra unite, progressively raising the emotional temperature to boiling point. Then the organ adds its enormous voice, uniting 1000 performers and listeners into a climactic proclamation of optimism: ‘Rise again, yes, rise again, Will you, my heart, in an instant! That for which you suffered, To God shall it carry you!*

## What is music?

The late Anthony Storr, psychiatrist, musician, and scholar, answered this question so well that we can do no better than précis and quote from his analysis. ( Storr, 1992) According to 6 ^th^century Roman philosopher Boethius, ‘Music is so naturally united with us that we cannot be free from it even if we so desired.’ Sound gives infants in the womb their first signal that they are part of a world that exists outside themselves. Music, Storr suggested, originated from ‘prosodic exchanges between mother and infant which foster the bond between them’. Music derives from ‘a subjective, emotional need for communication with other human beings which is prior to the need for conveying objective information or exchanging ideas.’ Music, in one form or another, is part of every culture. It is predominantly a group activity. The ability of music to ‘form communicative bonds between people makes it one of the fundamental activities of mankind’.

Music is a fundamental part of being human because it ‘can penetrate the core of our physical being. It can make us weep or give us intense pleasure. Music, like being in love, can temporarily transform our whole existence.’ Music is set apart from other forms of communication because it is non-propositional. ‘It does not put forward theories about the world or convey information in the same way as does language.’ ‘Absence of external association makes music unique amongst the arts’. And yet the ability of music to arouse strong emotions argues against it being a ‘disembodied system of relationships between sounds.’ Musical structure brings sounds together to form a sensory language.

Like visual art, the language of music can evoke responses in people, as opposed to communicating conceptual thought and proving objective relationships. In that regard, it is like metaphor, and anthropomorphic, embodied, and visual forms of communication. But the communal nature of music distinguishes it from other essentially subjective art-forms. When a group of people sing together, ‘a culturally agreed-upon pattern of rhythm and melody .. provides a shared form of emotion that, at least during the course of the song, carries along the participants so that they experience their bodies responding emotionally in very similar ways.’ Other art-forms, of course, are communal, but which of them brings people into such synchronous connection that their pulses speed up and slow down together, and they burst into expressions of emotions as one?

If objective evidence is needed to support Storr’s deeply subjective analysis, neurologist Oliver Sacks provided it. (Sacks, 2007) In an intriguing collection of case studies, he examined how neurological injury and dysfunction can produce or influence musical experience and behaviour. The first of these, which gives the book its title ‘Musicophilia’, describes a doctor who was hit by lightning, suffered cardiac arrest, and had a near-death experience. Soon afterwards, he developed a compulsive craving for piano music, which became a central part of his life, despite having had almost no part before. He was no crackpot. He carried on his medical practice, albeit with a very different approach, whilst devoting his life to learning and eventually performing technically demanding piano music. A striking feature of Sacks’ account is the rational and insightful way the musician-doctor spoke about this bizarre phenomenon. Other of Sacks’ case studies describe how musical forms or sounds can provoke complex partial seizures (temporal lobe epilepsy). A striking feature of these stories is the highly organized and socially constructed nature of the musical sound or activity that is so strongly linked to brain (dys)function. The temporal lobe of the brain and its connections are implicated. Sacks’ case studies support Storr’s contention that music, as we know it, connects with very fundamental neuro-psychological features of the human condition.

Returning now to our personal experiences, we find music relevant to medicine because it involves all our senses and connects us with other people as a whole. But what does this type of experience have to do with clinical practice? Is the notion of ‘tuning in’ to a patient a glib metaphor or is there something deeper in it? If music is about ‘being with’ other musicians and listeners rather than ‘doing music to them’, what does that tell us about medicine?

## Medicine: Tim’s story

*Aoife (not her real name) loved fun. Unbridled fun and diabetes don’t mix well, though, so the nerves to her blood vessels failed. At first, we laughed together when I juggled doses of powerful drugs so she could get out of a chair without fainting. As time went by, though, she couldn’t even sit up. Technical medicine had nothing more to offer. When laughter turned to tears, I asked for the first time how I could help. ‘Just be there’ was her answer. Aiofe taught me something simple but profound: doctors can only sometimes help by ‘doing things’ but they can always help by ‘being there’. This is nicely captured by the maxim: ‘Don’t just do something; stand there’.*

## Medicine: Martina’s story

*What has music to do with being a doctor? For me, it’s about that total engagement with another in a moment. We talk a lot in medicine about listening to the content of people’s speech; but what about tone, timbre, pauses, silence, breath, associated body movement, eye contact, touch, and smell, which are vital to how we interact with each other? Listening is not content, it is intense presence. To develop the analogy, before I see someone, I like to pause, to just focus on the person. That pause doesn’t even take a moment, but It’s like gathering oneself before the start of the performance. When I am with a patient I try to pay attention to everything; what they say, how they say it, what they don’t say, sometimes anticipating. I try to follow their tune and react. Even if it’s a well-rehearsed encounter, it is never quite the same. There is a movement to the interaction; something about ‘the live performance’ that resonates with how, as doctors, we interact with patients.*

## What is medicine?

Psychotherapist and general practitioner Michael Balint said that the most powerful drug in a physician’s pharmacopoeia is the physician themself. ( Balint, 1963) Ronald Epstein, a physician and practitioner of mindfulness, meticulously explains the ‘pharmacology’ of Balint’s ‘doctor as drug’ in his book ‘Attending’. ( Epstein, 2017) It is perhaps no coincidence that the book provides such rich answers to our question about the nature of medicine because Epstein’s first study was music. Alongside practising medicine, he plays that most tactile and subtle instrument, the harpsichord.

Epstein’s central thesis is that an ability to connect intimately with their own thoughts, feelings, and bodily sensations, in the moment, allows doctors to form therapeutic connections with patients. In the hands of mindless physicians, even the most sophisticated technologies lose therapeutic potency because mindful interpersonal connection is the essence of good medical practice. Patients trust mindful doctors because they feel understood, connected, and emotionally supported. The paragraphs that follow summarise key points from Epstein’s book. ( Epstein, 2017)

Mindful practice means being self-aware, monitoring oneself, and regulating one’s internal reactions and behaviour. It means being ‘empty’, so we have space within ourselves for new experiences and ideas. It means noticing our physical and emotional responses to patients and, rather than pulling back from difficult situations, being curious about them. Mindfulness creates conditions to respond empathically - in a bodily, emotional, and cognitive way to other people’s emotional lives. Epstein describes his habit of pausing, briefly, before entering any patient’s room. Taking a breath helps him be more present, mentally set aside the previous patient and other events of the day, let go of expectations, and be fresh and available.

Mindful doctors retain an ability to be naïve, even when expert. This is important because doctors can too easily be blind to important features of patients that are ‘hidden in full sight’. Mindfulness means paying focused attention to important features of clinical situations and maintaining ‘soft vigilance’; open, relaxed awareness of what is new, unexpected, or interesting. It means using our awareness of our inner state to helps us recognize the inner states of others. It means living each moment in the moment, knowing that our understanding of what we experience is always incomplete.

Mindful doctors see each patient as a person and turn towards their individual suffering. They listen deeply and are ‘present’. They don’t just observe, they see. They connect with patients verbally and non-verbally with a handshake, a softness of gaze, and by conducting physical examination gently. Mindful presence is slowing down, listening more deeply, thinking more deliberately, shifting from doing to being, and from activity to stillness.

Epstein’s description of the impact of mindful medicine - that patients are captivated, entranced, and transported, that time seems to stand still, and that they feel honoured and respected - is uncannily reminiscent of William Osler’s legendary bedside manner. He was ‘surrounded by such a distinctive, attracting, personal aura. It was something that was felt as well as seen. He had a knack of giving you all his attention and interest, perhaps taking you by the arm, listening intently, remarking on an encounter years earlier or on some other bond you had in common, convincing you that for William Osler at this moment you are the most important person in the world.’( Bliss, 1999)

## What do music and medicine have to do with one another?

Epstein’s analysis ( Epstein, 2017) shows how ‘doctor as drug’ stands apart from all other drugs. Rather than doing things to sick people, the mindful doctor is *with* sick people. Medical practice is, in that sense, existential rather than instrumental. We ‘tune in’ to patients, just as we tune into fellow musicians. In the same way, we experience music holistically. It engages all our senses. We use those senses to be in intimate communion with fellow musicians and listeners. Music unites all parties into a shared experience of being alive in the world. The union is founded on emotions and embodied and non-propositional experience as much as it is founded on the propositional logic of words and numbers. The union is intimate and synchronous. It takes us to heights of experience that, alone, we could not reach.

It is surprising, though, how little curiosity the medical profession shows towards the kinship between music and medicine, apart from making the ‘epidemiological’ observation that many doctors are musicians. In all my (Tim) experience of making music with doctors, I can’t remember a fellow medical musician talking about how the two practices link. The musical profession, in sharp contrast, is very aware of links between music and health. Our own research prompted musicians to reflect out loud by asking ‘how can music heal’? We analysed their responses using interpretive phenomenology. ( McLellan *et al.*, 2013) They said music could, holistically, improve the health of even seriously diseased people. It formed social links that made people feel less isolated. It could lift people’s moods and bring the spiritual, mental, and physical elements of their lives into better balance. A ‘transport’ metaphor - the ability of music to ‘take people to better places’ - epitomised many participants’ experiences. Music and mindful medicine have a lot to do with one another. Both of them create a type of social communication that cannot be described in words, which transports even sick people to better places.

## Using the senses to ‘perform’ medicine

A renowned cardiologist, Dr W Proctor Harvey, introduced medical students to auscultation by playing Beethoven’s symphonies. ( Van Drie, 2013) Afterwards, he asked if they had heard the music - of course, all put up their hands. But, when asked how many had heard the French horn or kettle drum, students put their hands down. Harvey used this simple exercise to demonstrate the need to listen attentively within human beings’ complex acoustic space for isolated sounds. Listening to the body is not easy, especially when, as a beginner, you don’t even know what you are listening for! ( Rice, 2010) Yet our ears learn to discern harmonies of health amidst the soundscape of the body just as our eyes learn to distinguish signs of disease from patterns of normality.

Perhaps you can recall the first time you walked in your gleaming white coat through hospital wards with a sense of nervous anticipation. The swish of curtains, the creak of bedside tables, trolleys pushed along the corridors, beepers beeping, phones trilling, voices of authority arousing fear. You approach your first patient, anxious to listen to their heart. Disappointingly, it is more or less impossible to hear heart sounds, let alone isolate the whoosh of aortic stenosis effortlessly identified by your preceptor. Instead you hear your own heart pounding as you follow the hands of your tutor as they describe the sound - hands up (crescendo), hands down (decrescendo); being musically trained, interestingly, helped students interpret the soundscape. ( Harris and Flynn, 2017)

Years later, the sounds of the hospital ward, operating theatre, or emergency room are so familiar, that you no longer hear them. They are the ‘white noise’ or background rhythm, of your day-to-day life. As you listen to the lub-dub of the heart, you hear the musical wheeze of trapped air. You no longer struggle to communicate the sounds you hear. You express yourself using a standardized professional terminology of grade 3/6, mid-systolic click, added fourth heart sound, and so on.

Becoming expert can, however, be at a cost. Attuning our ears to heart sounds heard through a stethoscope might desensitise us to more subjective sounds of sickness: human frailty, vulnerability, and distress. Closing our eyes or staring vacantly into the distance, absorbed by scientific sound, takes us to a diagnostic place where we feel safe but our patients have no voice. ( Baron, 1985; Rice, 2008)

Music comes into its own here. A skilled performer is every bit as attuned to other people’s playing as their own. And attuned to phrasing, articulation, and dynamics as well as whether the notes are right. Just as musical performance is an embodied communication with other musicians and listeners, so too does medicine engage all the senses of performers and listeners. Skilled diagnosticians do not isolate sound from other sensory experiences; rather their heightened listening is integrated with their other senses. ( Maslen, 2016) Anna Harris described this beautifully in her ethnographic study of learning to play percussion instruments. Is this, participants debate, a skill of listening or of touch?( Harris, 2016) It is both. In the same way, expert clinicians assimilate sight, sound, smell and touch into an integrated experience of patients’ illnesses. This includes the patient’s posture, facial expression, sound of breathing and pulse. Their proficient physical examinations are co-ordinated and smooth - their physical responses to their patients are orchestrated movements of body and mind as a form of bodily understanding. ( Merleau-Ponty, 1962) Whilst listening and hearing, touching and feeling, clinicians’ objective and subjective experiences merge in a sensescape of healing. ( Classen and Howes, 2006)

## Conclusions

Music and medicine, we have argued, are states of being, in the moment, with other people. Musicians do not ‘do’ music to their audiences, but engage in simultaneous, two-way communication that can transport both parties to better places. We have argued that medicine is, likewise, an act of being there, whether or not doctors do technical things to patients. The practices of medicine and music have subjective, non-verbal, dimensions that can engage all the senses. These dimensions, which are present in all cultures and societies, form connections that are deeply located in the human psyche. Good doctors, like good musicians, tune in to patients ‘in the moment’, and allow their own beings to connect with patients’ beings. Music can help students develop auditory skills but much work remains to be done on how it could make a non-instrumental, existential, contribution to medical education. This, we suggest, could help students and doctors reflect on their experiences of being in the world, and how shared experience can relieve suffering.

## Take Home Messages

• Music and medicine both involve ‘being present’
• Presence involves all a doctors’ senses, just as it involves all a musician’s senses
• There are close similarities between music and medicine, as communicative practices
• Music helps develop listening skills
• Listening is paying attention to how people speak as well as what they say
• Music, we suggest, could help doctors reflect on how the experience of ‘being in the world’ can relieve suffering

## Notes On Contributors

Tim Dornan is an internist and endocrinologist. He is Professor of Medical Education at Queen’s University Belfast, Northern Ireland, UK. His research interests are workplace learning and clinical humanism. He provides the perspective of a bassoonist, who plays in orchestras and chamber groups.

Martina Kelly is a family physician. She is Associate Professor at University of Calgary, Alberta, Canada. Her research interests are in embodiment and sensory learning, with a particular focus on touch. She provides the perspective of an engaged listener to an eclectic range of musical genres, and of a broader interest in medical humanities
