# Supplementary material for: Music and Medicine: being in the moment
Source: MedEdPublish (2016). 2018 Sep 5;7:197. [Version 1] doi: 10.15694/mep.2018.0000197.1 (PMC10701799; doi:10.15694/mep.2018.0000197.1)
Supplement: Peer review history

# Reviewer report: Reviewer response for version 1

**DOI:** 10.21956/mep.19658.r29332

**Published:** 2018-09-09

**Authors:** Jacek Mostwin

**Affiliations:** 1 Johns Hopkins Medical Institutions

This review has been migrated. The reviewer awarded 5 stars out of 5 I was in Mallorca when Dr. Dornan presented this unique and very stimulating paper, reminding us that, as with music, medicine is its own language. He played a simple piano recording: pure tones in a gothic room that until then had been filled only with spoken words. We were all affected by the change in mood, shaped by the pace and simple sounds of the composition. The experience of listening cannot be reproduced on these pages, and that was part of the point made from the podium, but a reader might select a favorite composition to play on a chosen instrument, or listen to it in a reflective moment to grasp the sense of what was shared with the conference audience. The authors can cover only so much in this brief paper, but their work encourages us to consider the many ways in which musical performance, training and appreciation parallels and affects the learning and practice of our own profession. They present a number of valuable ideas. But as with much of phenomenology, abstraction is not the goal. Rather, experience itself is at the heart of understanding, in that locus where “essence” is found. In a medical world currently dominated by linear reasoning that would convert subjective, intuitive and experiential dimensions into scalar quantities before permitting us to make sense of them, this appeal to musical intelligence and experience is refreshing. As they write in their text: "Rather than doing things to sick people, the mindful doctor is with sick people. Medical practice is, in that sense, existential rather than instrumental….The union is founded on emotions and embodied and non-propositional experience as much as it is founded on the propositional logic of words and numbers."

Reviewer Expertise:

NA

No decision status is available

# Reviewer report: Reviewer response for version 1

**DOI:** 10.21956/mep.19658.r29330

**Published:** 2018-09-05

**Authors:** P Ravi Shankar

**Affiliations:** 1 American International Medical University

This review has been migrated. The reviewer awarded 4 stars out of 5 This is an interesting and well written paper. I enjoyed reading and noting the development of ideas in the manuscript. I agree with the authors that music is less commonly used in the medical humanities. The visual and literary media are more commonly employed. The authors mention how doctors can help their patients simply by being there. A similar theme was developed in the article about the painting titled ‘The doctor’. Modern doctors and the healthcare system with its emphasis on efficiency and cost-utility analysis do not permit much time for doctors to simply ‘be there’. This could again be one of the factors which have contributed to the decline in patient confidence in doctors. I must confess that I still find it difficult to identify heart murmurs and a musical training may have better prepared me for clinical practice. Music and medicine both involve ‘being present’ as put forward by the authors. Humans have a number of constraints with regard to ‘being present’. Our minds dwell on the past or on the future and other factors impact our ability to be present in the moment. Meditation is another technic which will improve mindfulness and enable us to be more ‘present’ in the ‘present’. The article will be of interest to educators with a special interest in the humanities.

Reviewer Expertise:

NA

No decision status is available

# Reviewer report: Reviewer response for version 1

**DOI:** 10.21956/mep.19658.r29331

**Published:** 2018-09-05

**Authors:** Trevor Gibbs

**Affiliations:** 1 AMEE

This review has been migrated. The reviewer awarded 4 stars out of 5 An interesting paper to read and review that draws on the similarities between medicine and music. It would be hard to argue against the similarities of a listening musician and a listening doctor- of both of them being "there"- in "the moment". I enjoyed the picture that the authors drew of these similarities. From a cynical point of view, I might argue however that a "good" doctor might not come solely from being an understanding musician- however good is defined, nor perhaps an experienced musician would make a "good" doctor. Sadly my cynicism is based on knowing too many musicians who are far from the ideal when it comes to medicine and healthcare. I can however see the synergy between the two- we now need to know how to capture the shared qualities of the two and bring them together, whether inter or intra-curricula, and great our doctors of the future.

Reviewer Expertise:

NA

No decision status is available
